# Supplementary material for: Genome-Wide Association Studies of Biluochun Tea Plant Populations in Dongting Mountain and Comprehensive Identification of Candidate Genes Associated with Core Agronomic Traits by Four Analysis Models
Source: Plants (Basel). 2023 Oct 30;12(21):3719. doi: 10.3390/plants12213719 (PMC10650078; doi:10.3390/plants12213719)
Supplement: Supplementary file 1 [file plants-12-03719-s001.zip › Supplement figures.pdf]

Figure S1. Distribution of agronomic traits in Biluochun tea plant varieties. Red characters represented that frequency distributions of agronomic traits approximately meet normal distributions. Black characters represented that frequency distributions of agronomic traits do not meet normal distributions.

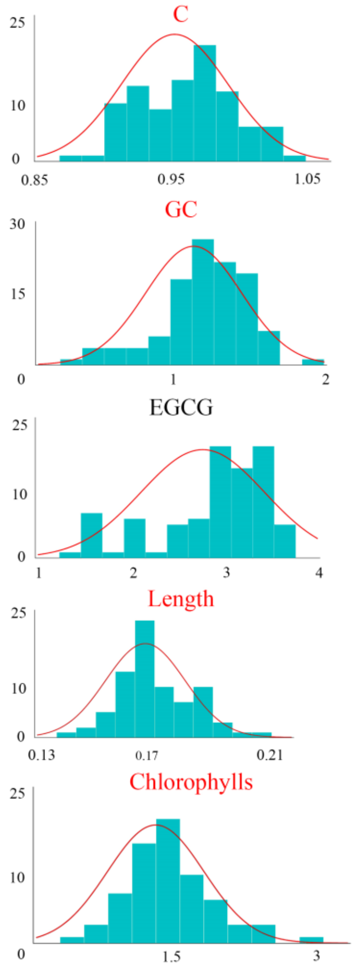

Figure S2. The correlation coefficient between pairwise metabolites, including SS, TP, TC, FAA, CAF, C, GC, GA, EC, EGC, ECG, CG, GCG, EGCG. Blue and positive numbers represent positive correlation, red and negative numbers represent negative correlation (* stands for p-value less than 0.05 and ** stands for *p*-value less than 0.01).

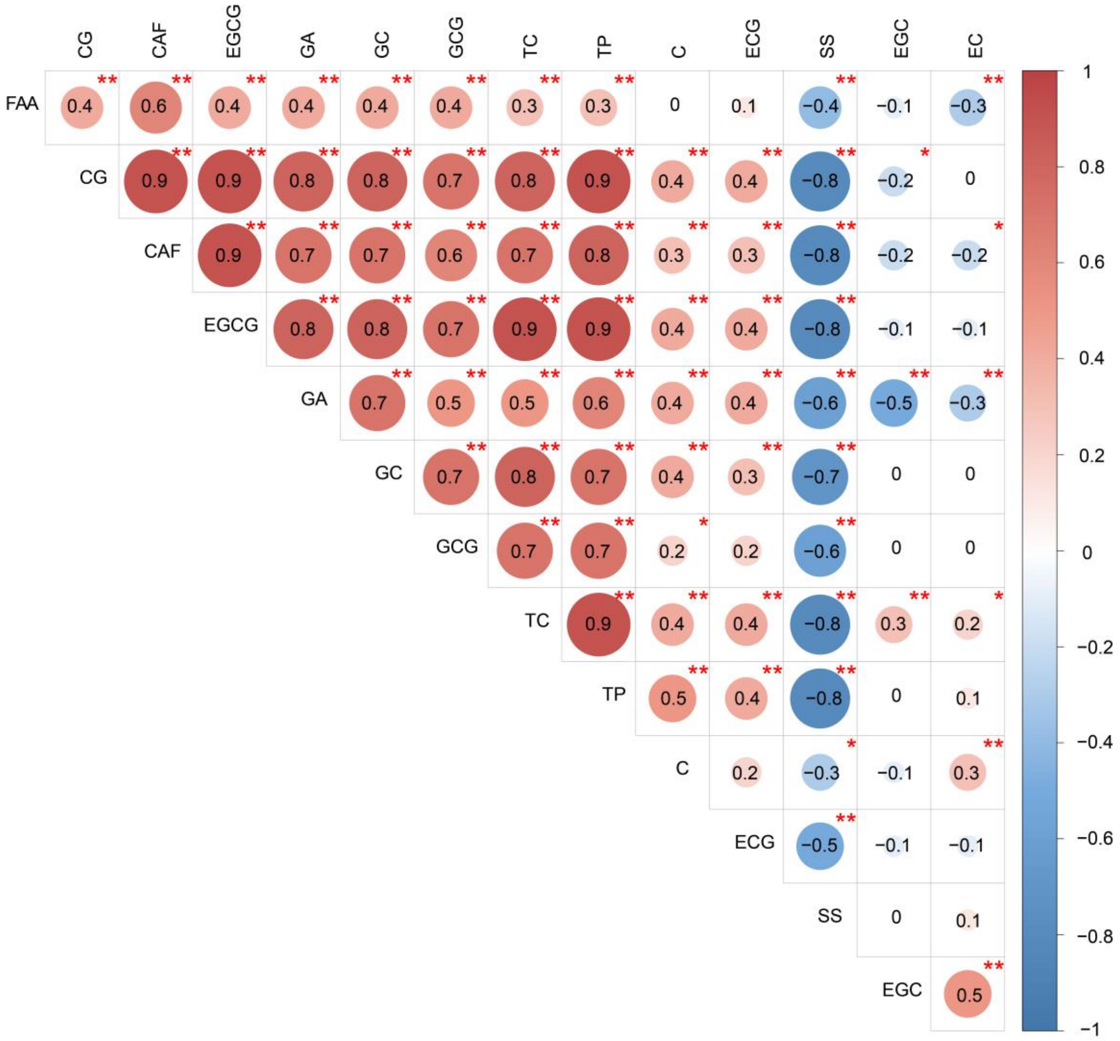

Figure S3. Population structure of 95 tea plant varieties. a. Cross validation of 95 Biluochun tea varieties from K=2 to K=9 clusters. b. Model-based Bayesian clustering of 95 Biluochun tea varieties.

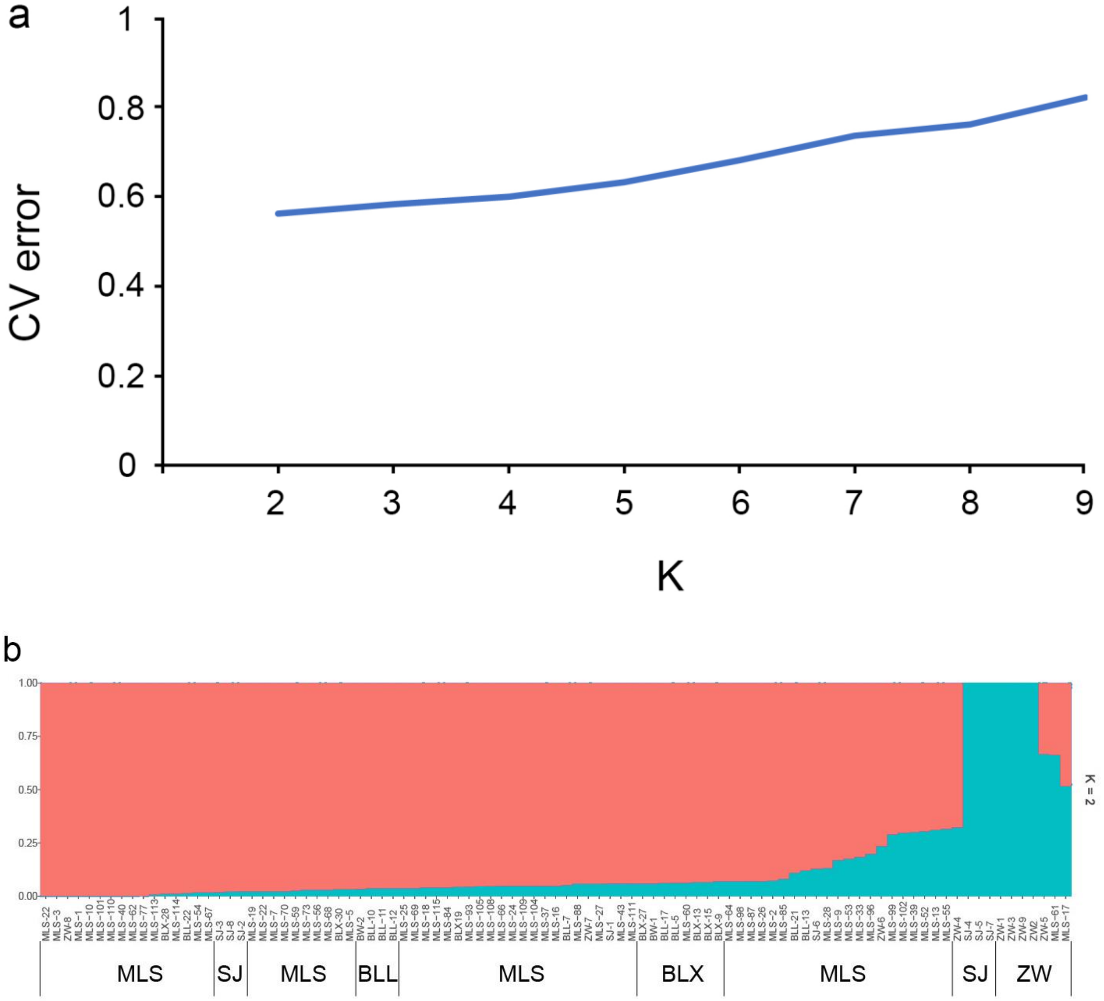

Figure S4. Distribution of SNP markers on chromosomes. The x-axis represents the number of SNPs in the 25 Mb window, and the y-axis represents 15 chromosomes of tea plant. Different colors represent different density of SNPs.

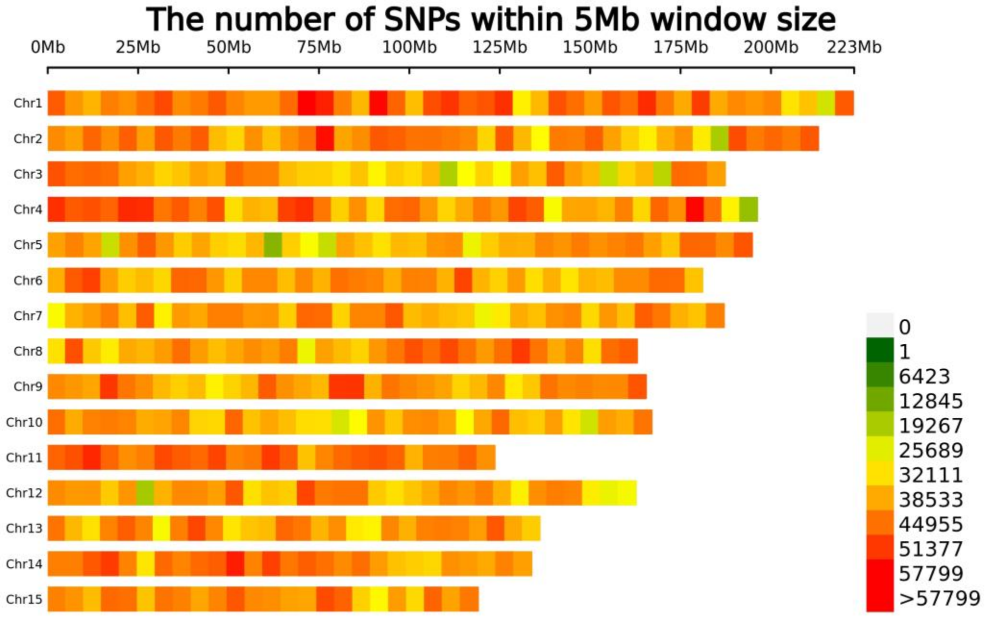

Figure S5. Manhattan and Quantile-quantile (QQ) plots of the GWAS for 14 characteristic traits. (A-N). Manhattan plots contain three phenotypic traits (leaf length, width and area); ten flavor-related metabolites (SS, FAA, TP, TC, C, EC, EGC, CG, ECG and EGCG) and a growth-related metabolite Chl. The x-axis displays the chromosome label, and the y-axis displays bonferroni correction threshold -log10 (*p*). The blue and red dashed lines show a significant correlation between SNPs and phenotypic values, with two thresholds p-value $< 3.89 \times 10^{-9}$ and p-value $< 7.78 \times 10^{-10}$, respectively. For quantile-quantile plots, the horizontal axis shows $-\log_{10}$ transformed expected *p* values, and the vertical axis indicates −log10 transformed observed *p* values. The red line represents the predicted value and the blue dot represents the observed value, which can show the difference between the predicted value and the observed value. Red, yellow, green, and cyan dots represent GLM, GLM(Q), MLM(K) and MLM(QK). (a-n) QQ plots represent the reliability of GLM analysis based on the above fourteen traits.

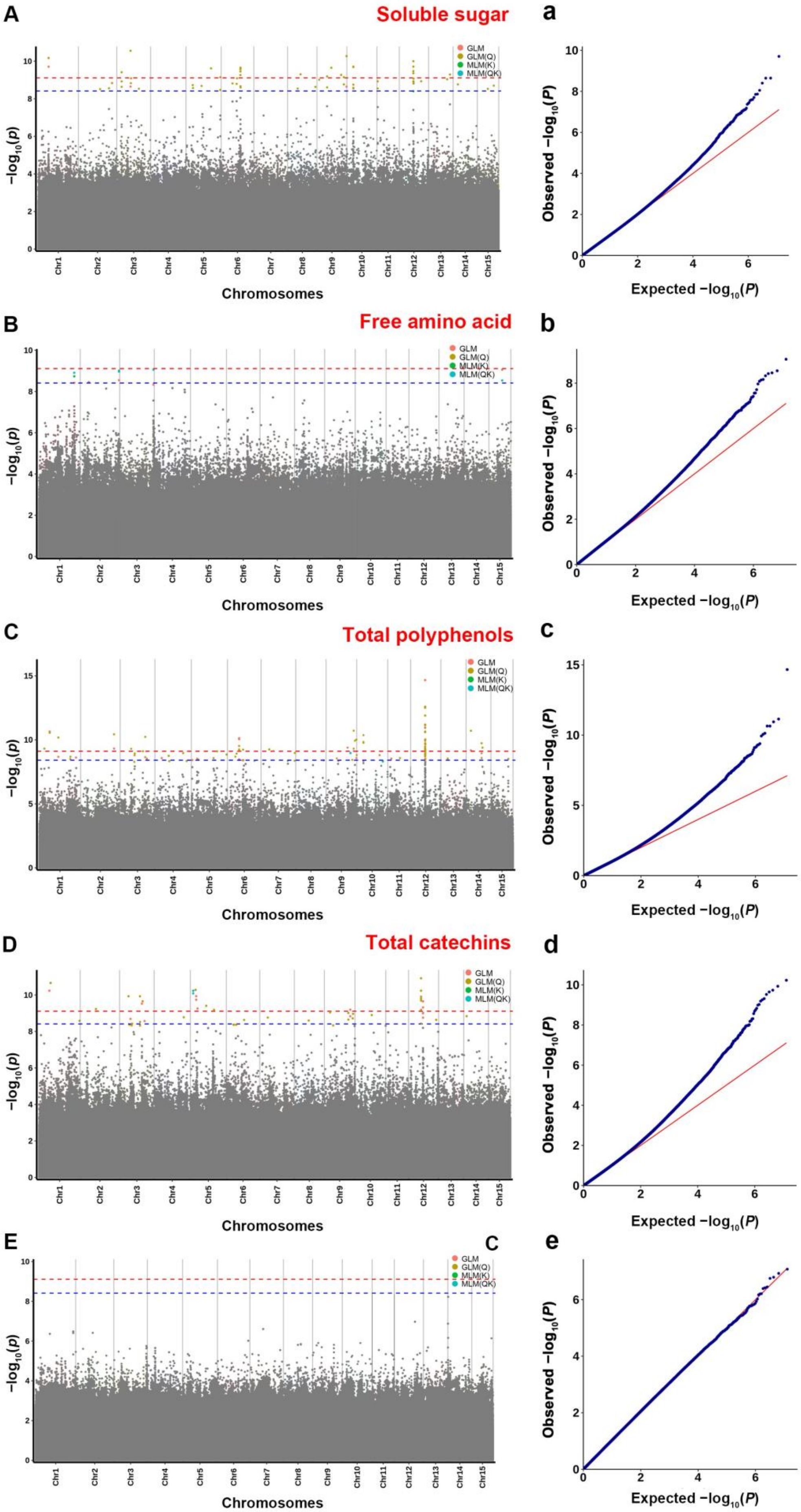
A
Soluble sugar
a
B
Free amino acid
b
C
Total polyphenols
c
D
Total catechins
d
E
C
e
GLM
GLM(Q)
MLM(K)
MLM(QK)
−log10(p)
Chromosomes
Chr1
Chr2
Chr3
Chr4
Chr5
Chr6
Chr7
Chr8
Chr9
Chr10
Chr11
Chr12
Chr13
Chr14
Chr15
Observed −log10(P)
Expected −log10(P)

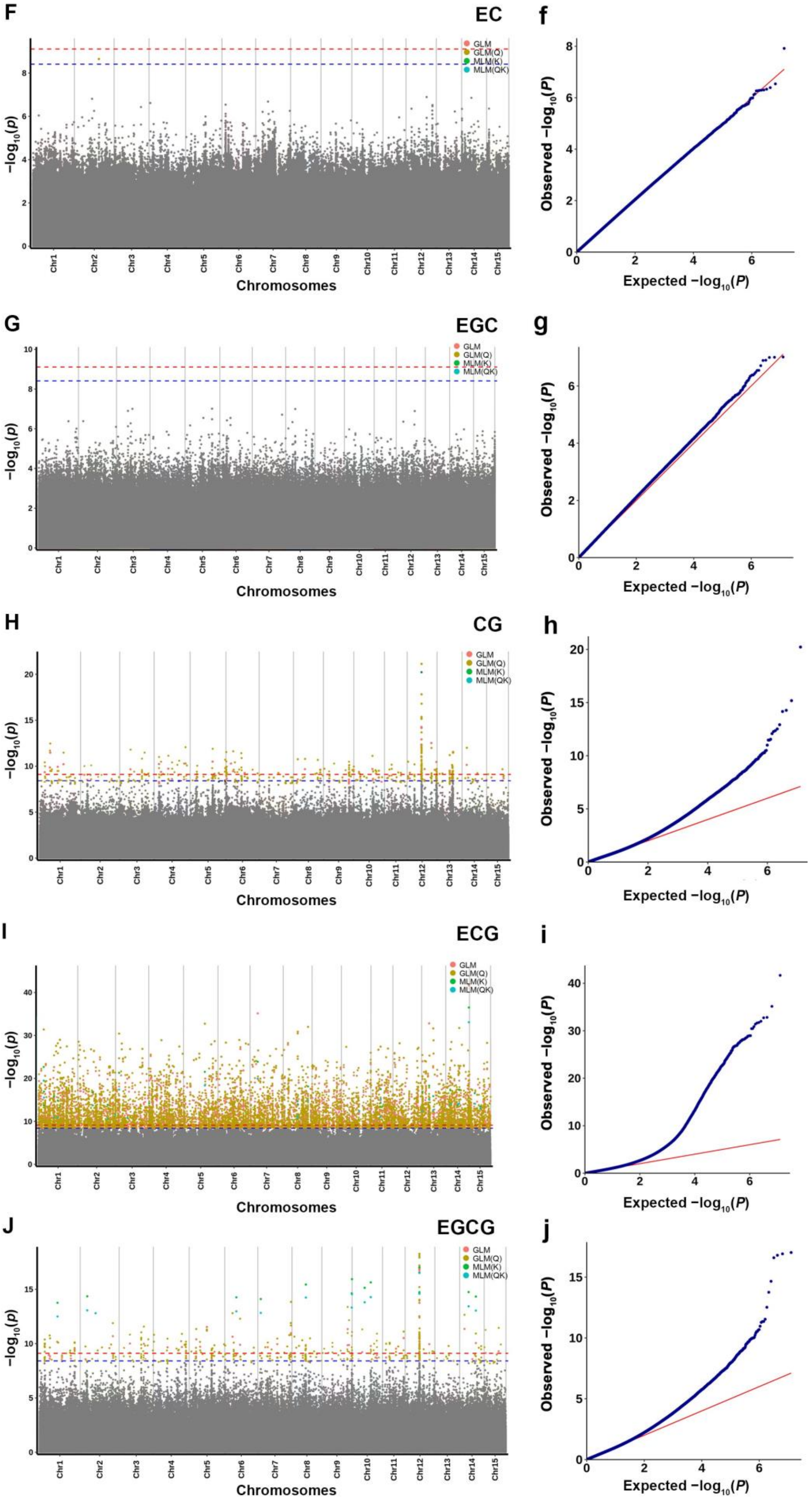
F
EC
GLM
GLM(Q)
MLM(K)
MLM(QK)
−log10(p)
Chr1 Chr2 Chr3 Chr4 Chr5 Chr6 Chr7 Chr8 Chr9 Chr10 Chr11 Chr12 Chr13 Chr14 Chr15
Chromosomes
f
Observed −log10(P)
Expected −log10(P)
G
EGC
GLM
GLM(Q)
MLM(K)
MLM(QK)
−log10(p)
Chr1 Chr2 Chr3 Chr4 Chr5 Chr6 Chr7 Chr8 Chr9 Chr10 Chr11 Chr12 Chr13 Chr14 Chr15
Chromosomes
g
Observed −log10(P)
Expected −log10(P)
H
CG
GLM
GLM(Q)
MLM(K)
MLM(QK)
−log10(p)
Chr1 Chr2 Chr3 Chr4 Chr5 Chr6 Chr7 Chr8 Chr9 Chr10 Chr11 Chr12 Chr13 Chr14 Chr15
Chromosomes
h
Observed −log10(P)
Expected −log10(P)
I
ECG
GLM
GLM(Q)
MLM(K)
MLM(QK)
−log10(p)
Chr1 Chr2 Chr3 Chr4 Chr5 Chr6 Chr7 Chr8 Chr9 Chr10 Chr11 Chr12 Chr13 Chr14 Chr15
Chromosomes
i
Observed −log10(P)
Expected −log10(P)
J
EGCG
GLM
GLM(Q)
MLM(K)
MLM(QK)
−log10(p)
Chr1 Chr2 Chr3 Chr4 Chr5 Chr6 Chr7 Chr8 Chr9 Chr10 Chr11 Chr12 Chr13 Chr14 Chr15
Chromosomes
j
Observed −log10(P)
Expected −log10(P)

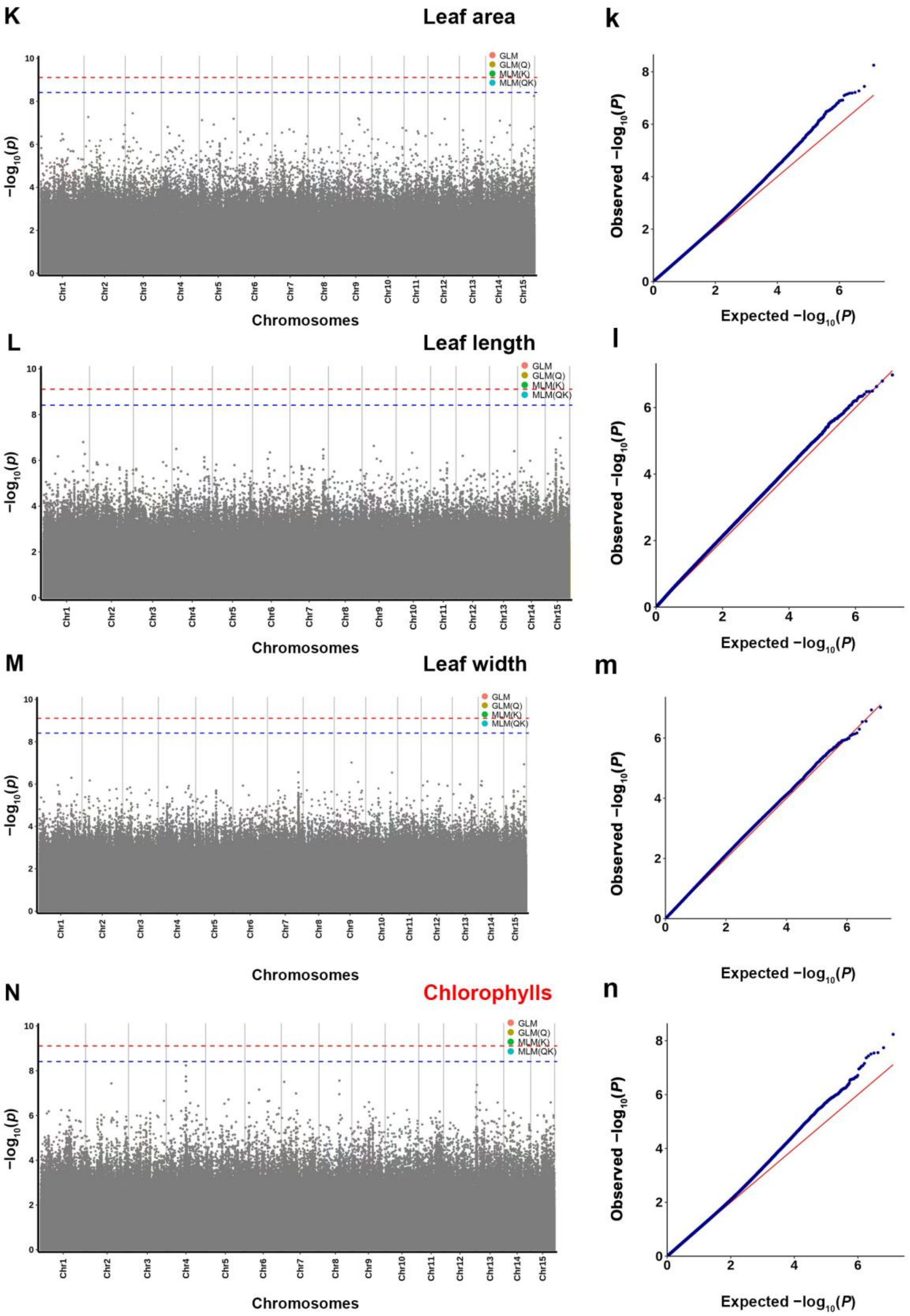
K
Leaf area
k
L
Leaf length
l
M
Leaf width
m
N
Chlorophylls
n
GLM
GLM(Q)
MLM(K)
MLM(QK)
−log10(p)
Chromosomes
Chr1
Chr2
Chr3
Chr4
Chr5
Chr6
Chr7
Chr8
Chr9
Chr10
Chr11
Chr12
Chr13
Chr14
Chr15
Observed −log10(P)
Expected −log10(P)

Figure S6. The sequence alignment of *CSS0039426*, *CSS0044862*, *CSS0026218* and *CSS0027172* related to GCG, GC and CAF and the distribution of nsSNPs in different haplotype types.

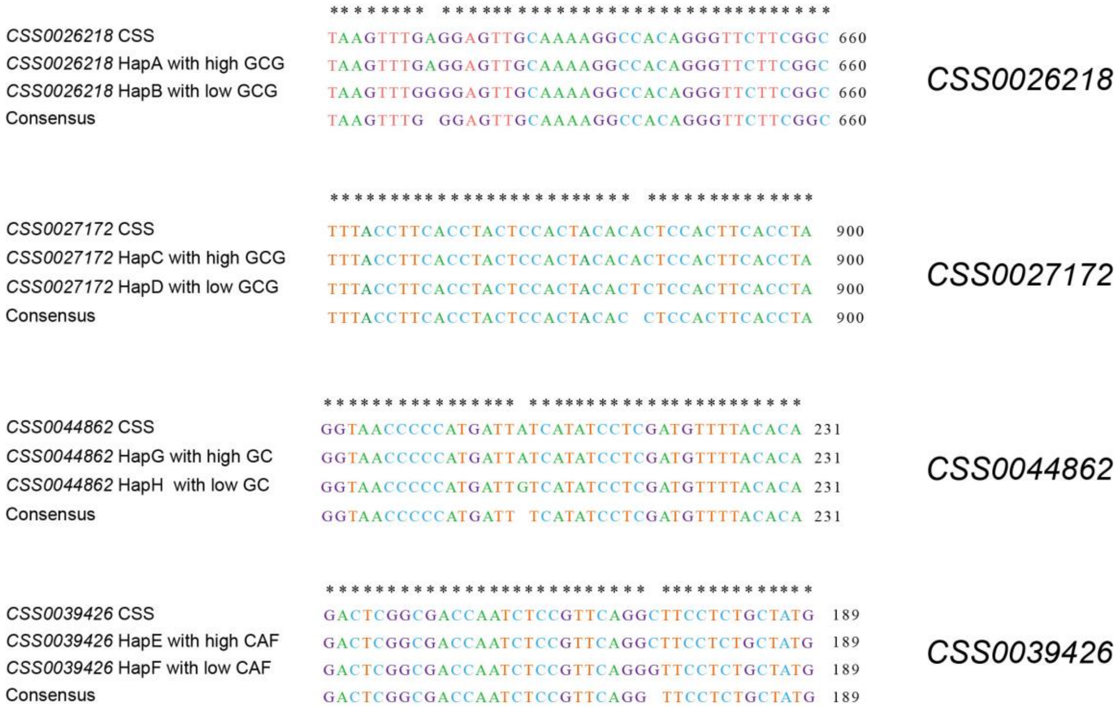

Figure S7. Box plot of GCG, GC and CAF content distribution about four pairs of haplotypes of *CSS0039426, CSS0044862*, *CSS0026218* and *CSS0027172*. The x-axis represents four pairs of haplotypes of *CSS0039426*, *CSS0044862*, *CSS0026218* and *CSS0027172* and the y-axis represents GCG, GC and CAF content. The below table is the detailed information of four pairs of haplotypes.

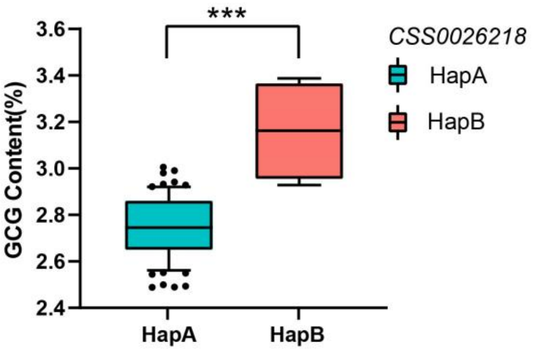

Haplotype	Position/bp	Chromosome	Mutation
HapA	164,656,928	9	A(Glu)
HapB			G(Gly)

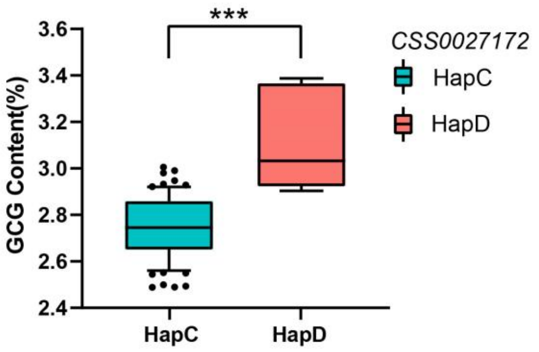

Haplotype	Position/bp	Chromosome	Mutation
HapC	140,146,183	9	A(Thr)
HapD			T(Ser)

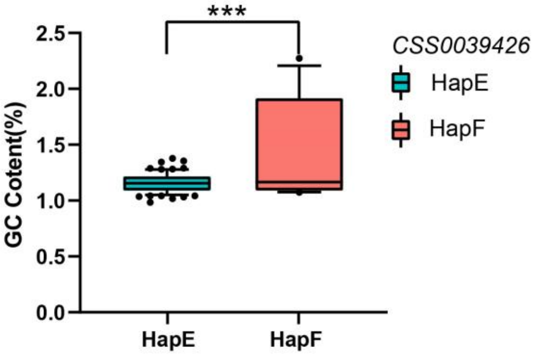

Haplotype	Position/bp	Chromosome	Mutation
HapE	141,418,417	9	A(Tyr)
HapF			G(Cys)

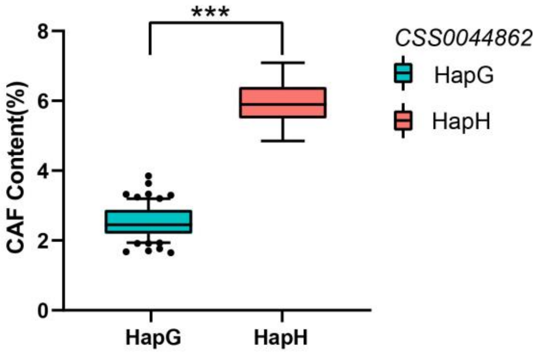

Haplotype	Position/bp	Chromosome	Mutation
HapG	144,566,106	9	C(Ala)
HapH			G(Gly)
